# A pilot study on the effect of three hydrocortisone dosing regimen on 17 hydroxyprogesterone levels in patients with congenital adrenal hyperplasia

**DOI:** 10.1186/1687-9856-2013-S1-P123

**Published:** 2013-10-03

**Authors:** Josephine Leiya Marie F Salud, Lorna Ramos-Abad

**Affiliations:** 1 University of the Philippines-Philippine General Hospital, Manila, Philippines

## 

The 2004 Newborn Screening Act has saved a lot of Filipinos from Congenital Adrenal Hyperplasia (CAH). The increase in the number of patients living with CAH challenges us to find the optimal steroid replacement therapy. In the Philippines, there is no locally published data on this. The objectives of the paper are to compare the effect of different hydrocortisone dosing regimen on the 17 hydroxyprogesterone (17 OHP) levels of patients with CAH and to report adverse effects.

The pilot study is a single-blinded randomized control trial comparing the 17 OHP levels of infants with CAH given higher evening hydrocortisone dose (HED), higher morning hydrocortisone dose (HMD) and equal hydrocortisone dose (EHD) who consulted in a tertiary hospital from January 2011 to July 2011. Nine patients completed the study (3 males and 6 females) with mean age of 16 (+11) months. One patient was randomized to HMD, 5 to HED and 3 to EHD. A marginally significant difference (p-value=0.0515) in the 17 OHP levels was observed between treatment groups. Adverse effects included increased BMI with concomitant presence of cushingoid facies in 33% (3/9) of the patients and an increased growth velocity in one patient in the HED group.

A significant difference was observed between treatment groups. The decrease in 17OHP was best appreciated in the EHD group but no definitive conclusion can be made on the optimal treatment schedule due to the small sample size. It is recommended that a larger study be done on newly diagnosed neonates with CAH using 525 patients (11 for HMD, 257 for HED and 257 for EHD) and ACTH levels be included in the outcome measures. Also, the use of oral steroids available in the country such as Prednisone or Dexamethasone as an alternative treatment be investigated.

